# Supplementary material for: Modelling human liver fibrosis in the context of non-alcoholic steatohepatitis using a microphysiological system
Source: Commun Biol. 2021 Sep 15;4:1080. doi: 10.1038/s42003-021-02616-x (PMC8443589; doi:10.1038/s42003-021-02616-x)
Supplement: Supplementary file 2 — Supplementary Information [file 42003_2021_2616_MOESM2_ESM.pdf]

Supplementary material

Modelling human liver fibrosis in the context of non-alcoholic steatohepatitis using a microphysiological system

Tomasz Kostrzewski[1], Sophie Snow[1], Anya Lindström Battle[1], Samantha Peel[2], Zahida Ahmad[3], Jayati Basak[3], Manasa Surakala[4], Aurelie Bornot[5], Julia Lindgren[6], Maria Ryaboshapkina[7], Maryam Clausen[6], Daniel Lindén[8,9], Christian Maass[10,11], Lucy May Young[1], Adam Corrigan[5], Lorna Ewart[3], and David Hughes[1].

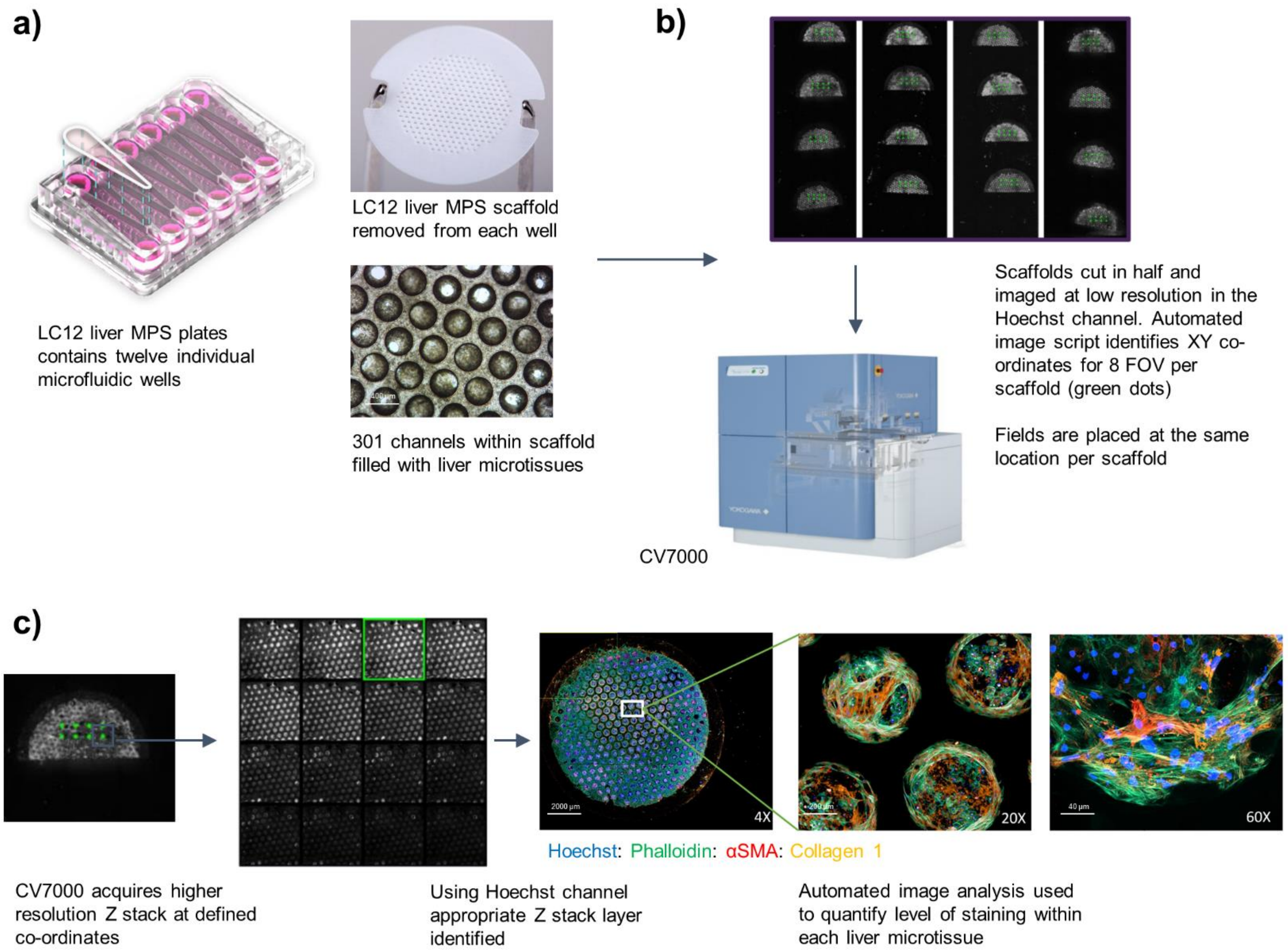

Supplemental Figure 1 – Automated imaging assay to determine fibrosis in NASH liver microtissues from MPS.

To assess the fibrosis phenotype in liver microtissues from the MPS an automated confocal imaging process was developed; a) Scaffolds containing liver microtissues were removed from each well of the liver MPS plate, with each scaffold containing an array of microtissues. Each scaffold was cut in half and then stained with antibodies against αSMA and collagen type-I. b) Half scaffolds were mounted on microscope slides and imaged by the Yokogawa CV7000. c) Low resolution images across the whole scaffold were taken and stitched together and then an automated script identified eight fields of view (FOV) on each scaffold and these were imaged at higher magnification and with a full Z-stack. The locations of the FOV were the same for all scaffolds in the experiment to exclude bias. The Hoechst (nuclei) channel was used to identify the Z-stack images for each FOV with the microtissue at the correct focus. Each microtissue and each FOV could then be analysed and quantified for the presence of the proteins of interest.

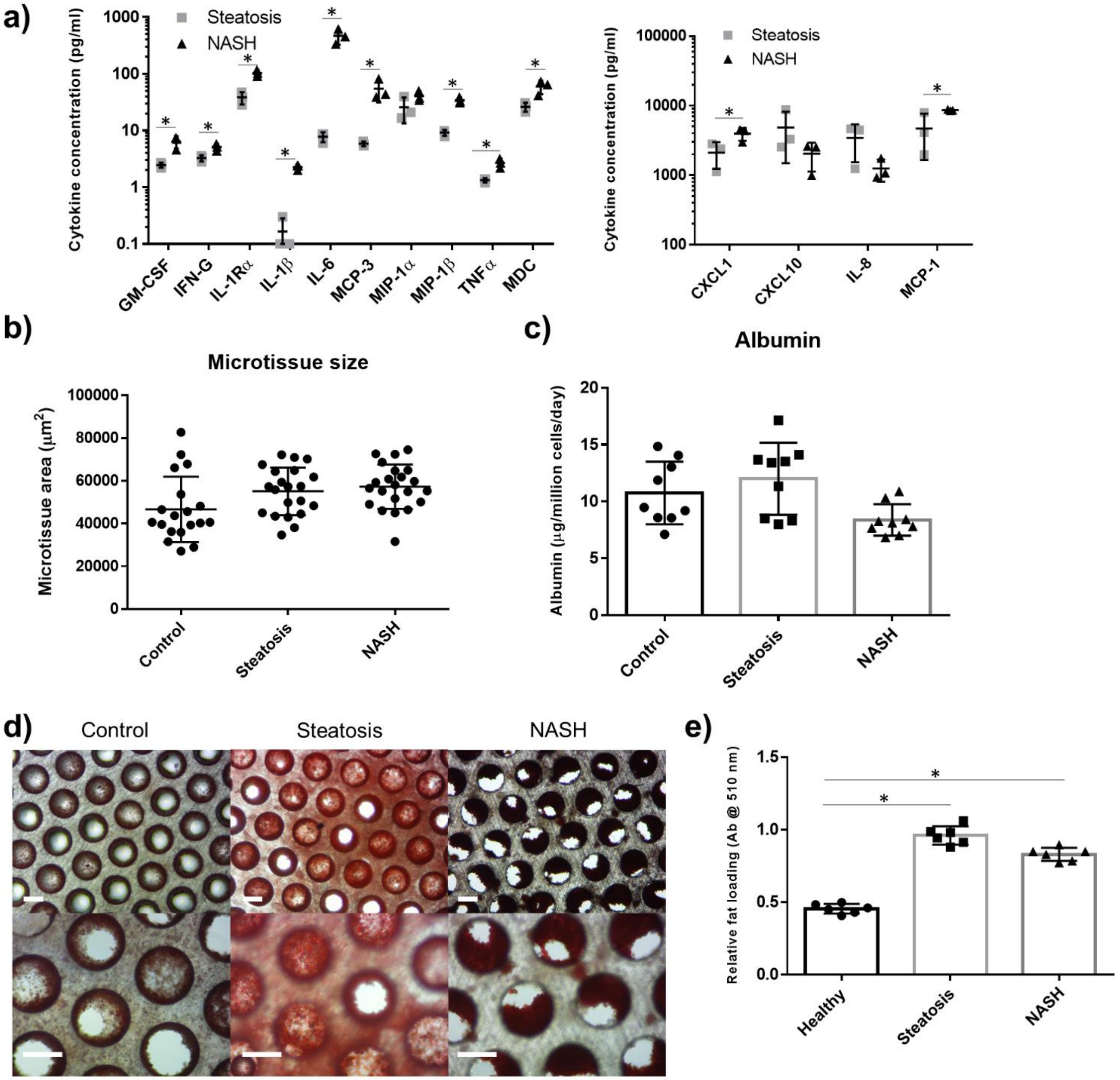

Supplemental Figure 2 – Liver MPS NASH model has an inflammatory and steatotic phenotype but does not display features of liver damage.

PHH alone (Control and Steatosis) or PHH, KC and HSC co-cultures (NASH) were cultured in the MPS platform for 14 days under standard media (control) or high fat conditions (Steatosis and NASH). a) Secreted inflammatory markers were all measured by Luminex in cell culture medium at the end of the culture. Control data not shown as most cytokines undetected. b) Size of microtissues from each sample was assessed by quantifying Hoechst staining in each FOV. c) Albumin production between conditions was compared by ELISA. d) Scaffold from all conditions were stained with Oil Red O to identify lipid loading, images were taken using brightfield microscope (scale bar = 200 µm), representative images shown and e) staining was quantified by destaining scaffolds and quantifying staining by absorbance @ 510 nm. All datapoints shown with error bars showing means ± SD. Data is from a minimum of nine independent cultures: P * < 0.05 determined by student T-test.

Gene symbol	Steatosis	NASH	Gene symbol	Steatosis	NASH	Gene symbol	Steatosis	NASH	Gene symbol	Steatosis	NASH
ACTA2	-2.04	-1.95	HGF	-1.84	-1.23	ITGB8	-2.04	-1.95	SMAD3	0.07	0.18
AGT	-1.59	-1.95	IFNG	-1.91	-1.55	LTBP1	-2.04	-1.95	SMAD6	0.20	-0.10
AKT1	-1.77	-1.46	IL10	-1.42	-1.40	MMP1	-2.04	-1.95	SMAD7	-0.10	0.18
BCL2	0.65	0.74	IL13	0.33	0.38	MMP14	-0.14	0.06	SNAI1	-2.04	-1.95
BMP7	-0.81	-0.50	IL13RA2	-2.04	-1.95	MMP2	-1.07	-0.30	SP1	-1.46	-1.67
CCL11	-1.28	-1.46	IL1A	-0.07	0.15	MMP3	-1.96	-1.85	STAT1	-1.15	-1.06
CCL2	-2.04	-1.95	IL1B	-2.03	-1.66	MMP8	-0.46	-0.07	STAT6	-0.60	-0.26
CCL3	-0.89	-0.52	IL4	-1.27	-1.51	MMP9	0.05	-0.53	TGFB1	-0.86	-0.61
CCR2	-0.20	-0.39	IL5	-1.25	-1.31	MYC	-0.41	-0.68	TGFB2	-1.90	-1.49
CEBPB	-0.15	-0.02	ILK	-1.11	-1.56	NFKB1	-0.80	-0.57	TGFB3	-1.46	-1.04
COL1A2	-2.04	-1.95	INHBE	-1.46	-1.72	PDGFA	-0.09	-0.12	TGFBR1	-0.25	-0.50
COL3A1	-0.92	-1.05	ITGA1	-1.73	-1.80	PDGFB	-1.73	-1.95	TGFBR2	0.32	-0.35
CTGF	-2.04	-1.95	ITGA2	-2.04	-1.95	PLAT	0.31	0.25	TGIF1	-1.53	-0.79
CXCR4	-1.81	-1.95	ITGA3	0.24	0.24	PLAU	-1.08	-1.00	THBS1	-0.05	-0.70
DCN	1.32	1.47	ITGAV	-2.04	-1.95	PLG	-1.83	-1.95	TIMP1	0.35	0.17
EDN1	-0.11	0.00	ITGB1	-2.04	-1.95	SERPINA1	-2.04	-1.59	TIMP2	-1.03	-1.62
EGF	-0.18	-0.32	ITGB3	-1.05	-0.64	SERPINE1	-0.24	-1.23	TIMP3	-1.23	-0.80
ENG	-0.47	-1.42	ITGB5	-0.96	-1.18	SERPINH1	-0.95	-0.55	TIMP4	-1.71	-1.95
FASLG	-0.59	-0.94	ITGB6	0.55	0.89	SMAD2	-1.56	-1.45	VEGFA	-1.28	-1.06

Supplemental Table 1 – Expression of fibrosis-related genes in steatosis and NASH MPS models.

PHH, KC and HSC co-cultures and PHH monocultures were cultured in the MPS platform under high fat conditions for 14 days and total RNA extracted and analysed by Human Fibrosis RT2 Profiler PCR Arrays. Gene expression levels are expressed as Log10 relative expression compared to housekeeping genes (GAPDH/B2M/HPRT1). Data are means of a nine independent cultures.

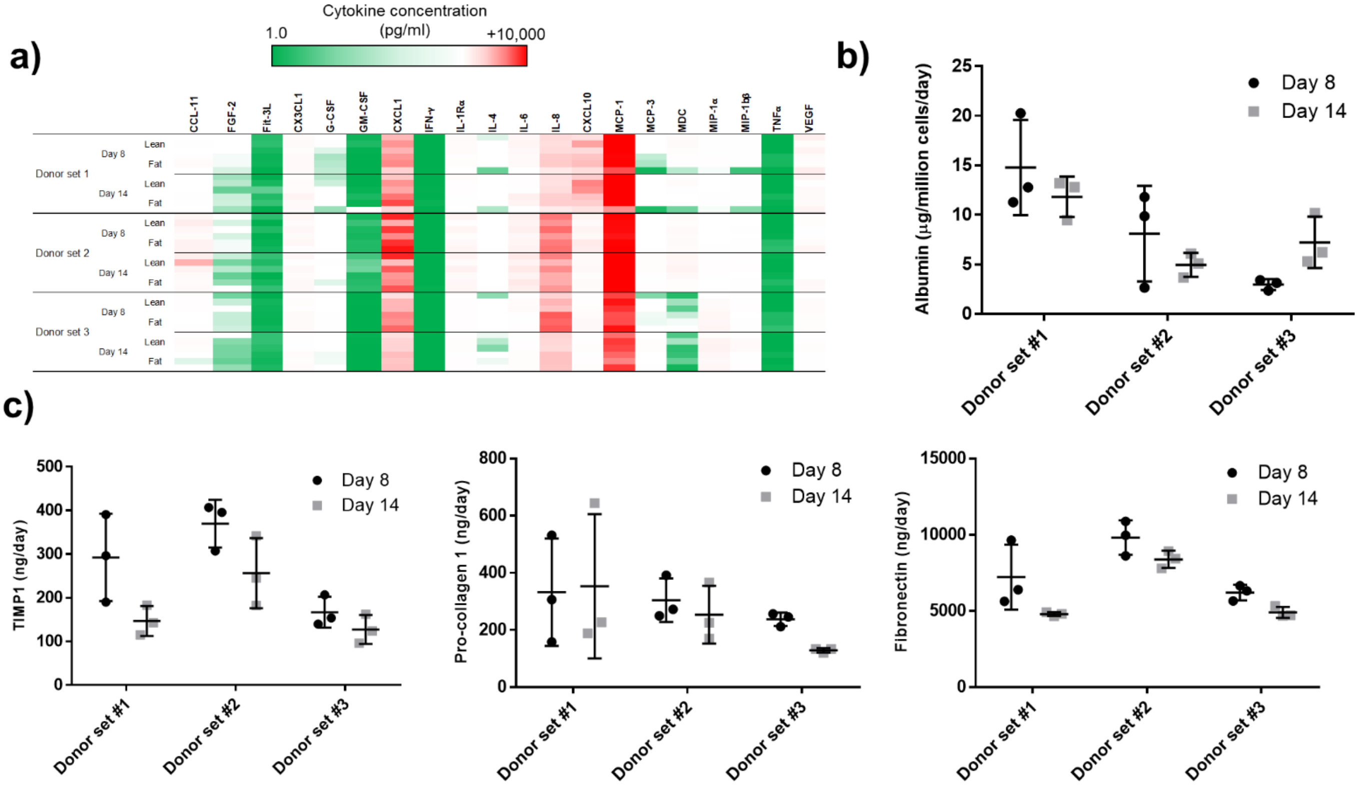

Supplemental Figure 3 – Liver MPS NASH model demonstrates minimal donor-donor variability.

PHH, KC and HSC co-cultures (with cells from different donors for each cell type) were cultured in the MPS platform for 14 days under lean or high fat conditions. a) Secreted inflammatory markers were all measured by Luminex in cell culture medium at day 8 and day 14 of the culture. Heat map shows mean absolute production of individual cytokines for each donor set. b) Albumin production between conditions was compared by ELISA. c) Secreted fibrosis markers were all measured by ELISA in cell culture medium at day 8 and day 14 of the culture. All datapoints are shown with error bars showing means ± SD. Data is from a minimum of three independent cultures.

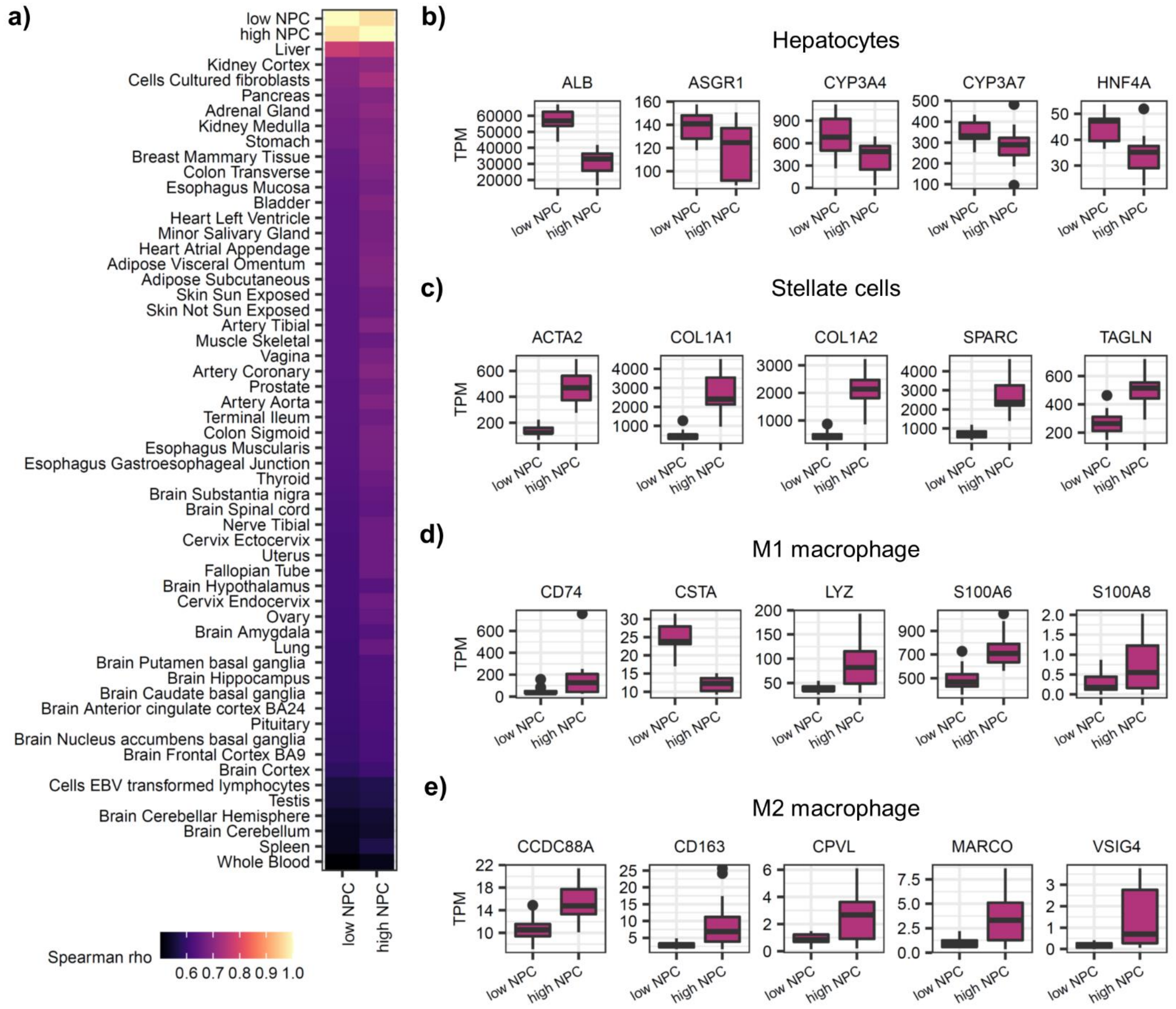

Supplemental Figure 4 – Transcriptional profile of control lean microtissues corresponded to normal human liver profile.

PHH, KC and HSC co-cultures (with varying NPC number) were cultured in the MPS platform for 14 days under lean conditions prior to RNA-seq analysis. a) Median transcripts per million kilobases (TPM) values for 18,492 genes were determined for low NPC and high NPC liver microtissues from MPS and compared to the expression in a variety of human tissues in the GTEx database (https://gtexportal.org/home/). Spearman correlation coefficients were calculated for all reliably detected genes with median expression > 0.5 TPM in the control lean microtissues or GTEx liver. Markers of specific cell types were assessed in same TPM data – b) mature hepatocytes, c) stellate cells and d-e) macrophages. Cell type markers were selected based on single-cell RNA-seq on normal human donor liver[70]. N=12 replicates for each condition.

ensembl_gene_id	hgnc_symbol	baseMean	ensembl_gene_id	hgnc_symbol	baseMean	ensembl_gene_id	hgnc_symbol	baseMean	ensembl_gene_id	hgnc_symbol	baseMean
ENSG00000163631	ALB	810178.5	ENSG00000137801	THBS1	59070.61	ENSG00000099194	SCD	34499.47	ENSG00000117984	CTSD	22549.07
ENSG00000198804	MT-CO1	575121.2	ENSG00000251562	MALAT1	55397.93	ENSG00000196136	SERPINA3	33471.06	ENSG00000106927	AMBP	22469.4
ENSG00000115414	FN1	462254.3	ENSG00000167996	FTH1	55302.13	ENSG00000138207	RBP4	31888.23	ENSG00000147872	PLIN2	22226.38
ENSG00000210082	MT-RNR2	382394.4	ENSG00000211459	MT-RNR1	53220.16	ENSG00000106366	SERPINE1	31333.45	ENSG00000185624	P4HB	22126.95
ENSG00000257017	HP	353330.5	ENSG00000243649	CFB	52872.25	ENSG00000198840	MT-ND3	31261.11	ENSG00000162267	ITIH3	21822.34
ENSG00000198886	MT-ND4	266152.9	ENSG00000175899	A2M	52423.14	ENSG00000120885	CLU	31134.63	ENSG00000250722	SELENOP	21671.18
ENSG00000171560	FGA	242181.7	ENSG00000173432	SAA1	52209.61	ENSG00000104760	FGL1	30715.77	ENSG00000182718	ANXA2	21638.99
ENSG00000108821	COL1A1	218581.3	ENSG00000112096	SOD2	51001.4	ENSG00000187134	AKR1C1	30285.81	ENSG00000150991	UBC	21090.81
ENSG00000197249	SERPINA1	194073	ENSG00000163359	COL6A3	50655.72	ENSG00000164733	CTSB	29976.78	ENSG00000047457	CP	20679.66
ENSG00000171564	FGB	174121.7	ENSG00000132693	CRP	50417.19	ENSG00000182326	C1S	29907.67	ENSG00000228278	ORM2	20584.77
ENSG00000198712	MT-CO2	173162.1	ENSG00000134339	SAA2	48015.48	ENSG00000109072	VTN	28970.2	ENSG00000145192	AHSG	20214.71
ENSG00000198938	MT-CO3	172159	ENSG00000145321	GC	46923.77	ENSG00000167658	EEF2	28175.01	ENSG00000161011	SQSTM1	19608.9
ENSG00000198727	MT-CYB	160648.4	ENSG00000283907		46113.78	ENSG00000118271	TTR	28073.69	ENSG00000159403	C1R	19556.13
ENSG00000171557	FGG	139728	ENSG00000168542	COL3A1	43939.68	ENSG00000196924	FLNA	27826.87	ENSG00000080824	HSP90AA1	19496.53
ENSG00000229314	ORM1	125549.4	ENSG00000113140	SPARC	43041.53	ENSG00000100345	MYH9	26965.52	ENSG00000026025	VIM	19246.02
ENSG00000156508	EEF1A1	121460.9	ENSG00000091513	TF	42301.79	ENSG00000198848	CES1	26611.72	ENSG00000130635	COL5A1	19197
ENSG00000198786	MT-ND5	111479.9	ENSG00000184009	ACTG1	42064.29	ENSG00000111640	GAPDH	26428.15	ENSG00000123384	LRP1	19173.44
ENSG00000125730	C3	104869.2	ENSG00000158874	APOA2	41352.35	ENSG00000000971	CFH	26270.2	ENSG00000224389	C4B	19096.06
ENSG00000198763	MT-ND2	103481.4	ENSG00000100234	TIMP3	39331.99	ENSG00000113889	KNG1	25063.18	ENSG00000164111	ANXA5	18939.18
ENSG00000164692	COL1A2	102970.1	ENSG00000091583	APOH	38232.25	ENSG00000179218	CALR	24732.87	ENSG00000166598	HSP90B1	18747.32
ENSG00000087086	FTL	85474.81	ENSG00000130203	APOE	36233.62	ENSG00000212907	MT-ND4L	23813.7	ENSG00000197746	PSAP	18628.04
ENSG00000198899	MT-ATP6	85189.77	ENSG00000136872	ALDOB	35131.14	ENSG00000124942	AHNAK	23382.49	ENSG00000044574	HSPA5	18447.11
ENSG00000084674	APOB	79312.74	ENSG00000133112	TPT1	35102.73	ENSG00000205542	TMSB4X	23179.01	ENSG00000122786	CALD1	18189.93
ENSG00000198888	MT-ND1	74842.7	ENSG00000150093	ITGB1	34930.23	ENSG00000196616	ADH1B	22841.73	ENSG00000149131	SERPING1	17528.68
ENSG00000075624	ACTB	66614.32	ENSG00000166710	B2M	34771.32	ENSG00000115306	SPTBN1	22665.85	ENSG00000171766	GATM	17405.46

Supplemental Table 2 – Top 100 genes expressed in NASH microtissues in MPS.

PHH, KC and HSC co-cultures were cultured in the MPS platform under high fat conditions for 14 days and total RNA extracted and analysed by RNA-seq to determine overall transcriptomic profile. Breakdown of top 100 genes in liver MPS NASH model.

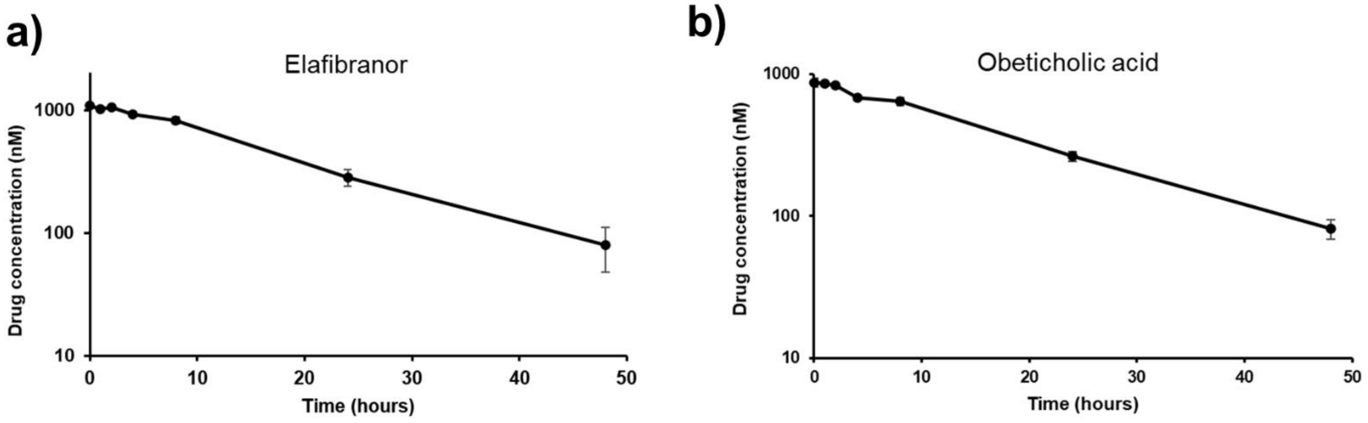

Supplemental Figure 5 – Obeticholic acid and Elafibranor are metabolised in the NASH MPS model.

The metabolism of a) Elafibranor and b) Obeticholic acid and was assessed in PHH, KC and HSC co-cultures grown in the liver MPS platform for 7 days. Following a double media exchange the compounds were incubated with the microtissues at a starting concentration of 1 µM and media samples taken at regular intervals across 48 hours. Media samples were assessed for the concentration of compound by quantitative LC-MS. Data show are mean ± SD from three independent cultures.

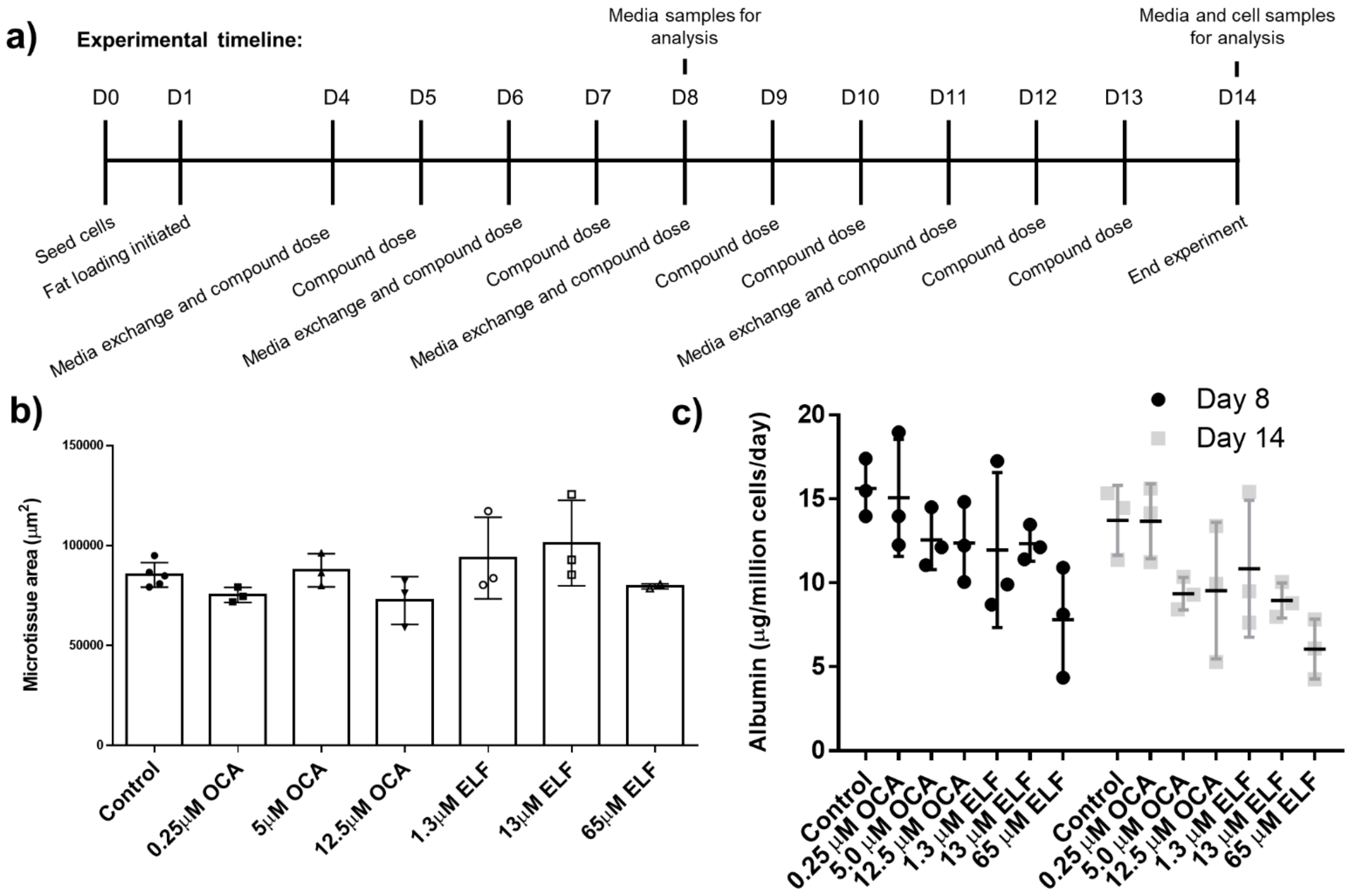

Supplemental Figure 6 – Treatment of NASH microtissues in MPS platform with Obeticholic acid and Elafibranor does not cause cytotoxicity.

PHH, KC and HSC co-cultures were cultured in the MPS platform under high fat conditions and dosed with varying concentrations of Obeticholic acid (OCA) and Elafibranor (ELF) dosed QD for 10 days, following an initial 4-day pre-culture phase. A) Overview of the experimental timeline. B) Tissue density of scaffolds from each control was assessed by quantifying Hoechst staining in each FOV (8 FOV per culture) at the end of the study. C) Albumin production between conditions was compared by ELISA at two timepoints. All datapoint are shown and error bars highlight means ± SD.

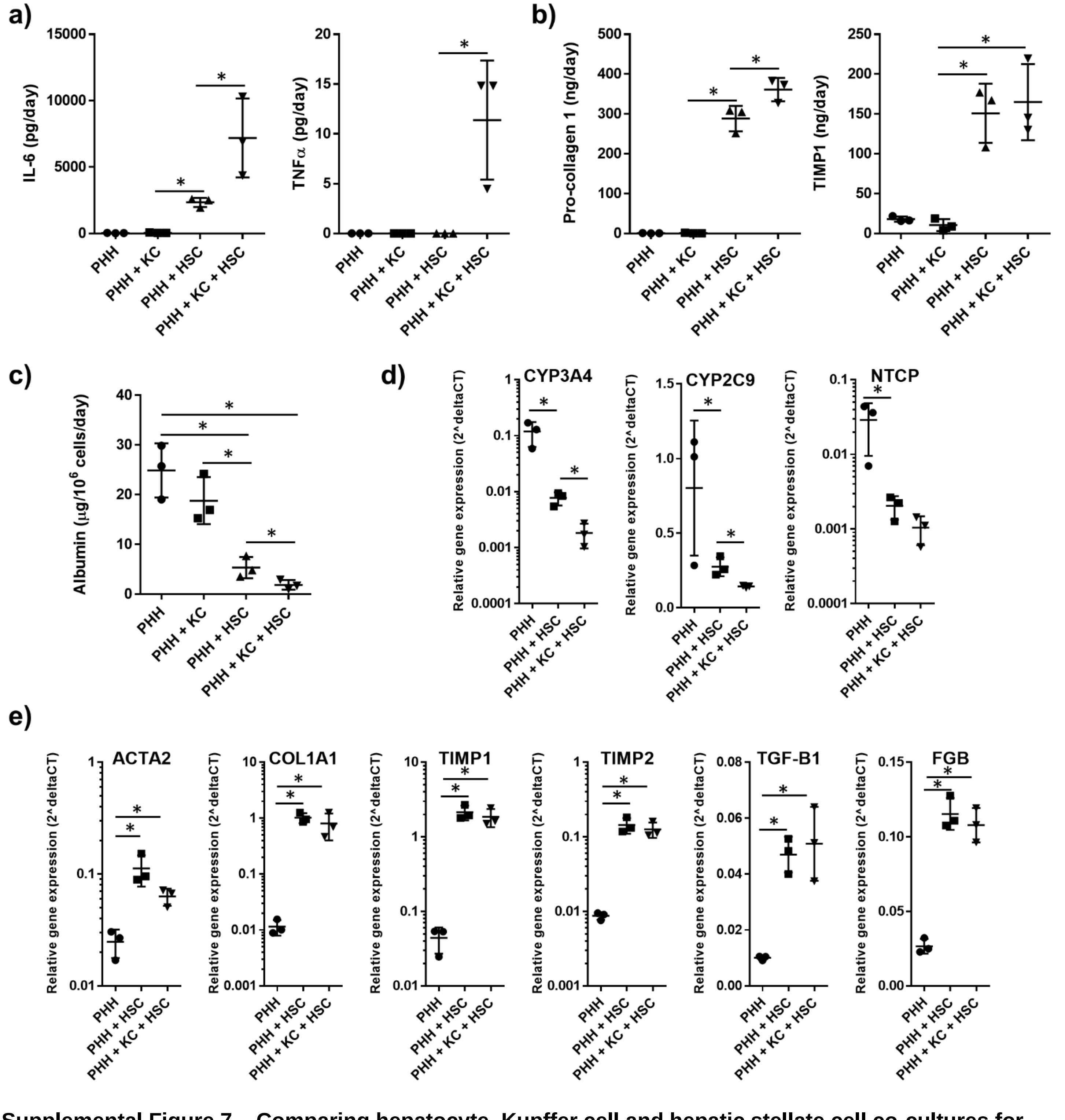

Supplemental Figure 7 – Comparing hepatocyte, Kupffer cell and hepatic stellate cell co-cultures for NASH phenotype.

PHH alone, PHH + HSC, PHH + KC or PHH, KC and HSC co-cultures were cultured in the MPS platform for 7 days under high fat conditions. A) Secreted inflammatory or B) fibrotic markers were all measured by ELISA in cell culture medium at the end of the culture. Control data not shown as most cytokines undetected. C) Albumin production between conditions was compared by ELISA. D) The expression of hepatic genes and E) pro-fibrotic genes was compared between conditions by QPCR and data expressed as relative expression compared to GAPDH housekeeping gene. All data are means ± SD from a minimum of three independent cultures: P * < 0.05 determined by student T-test.

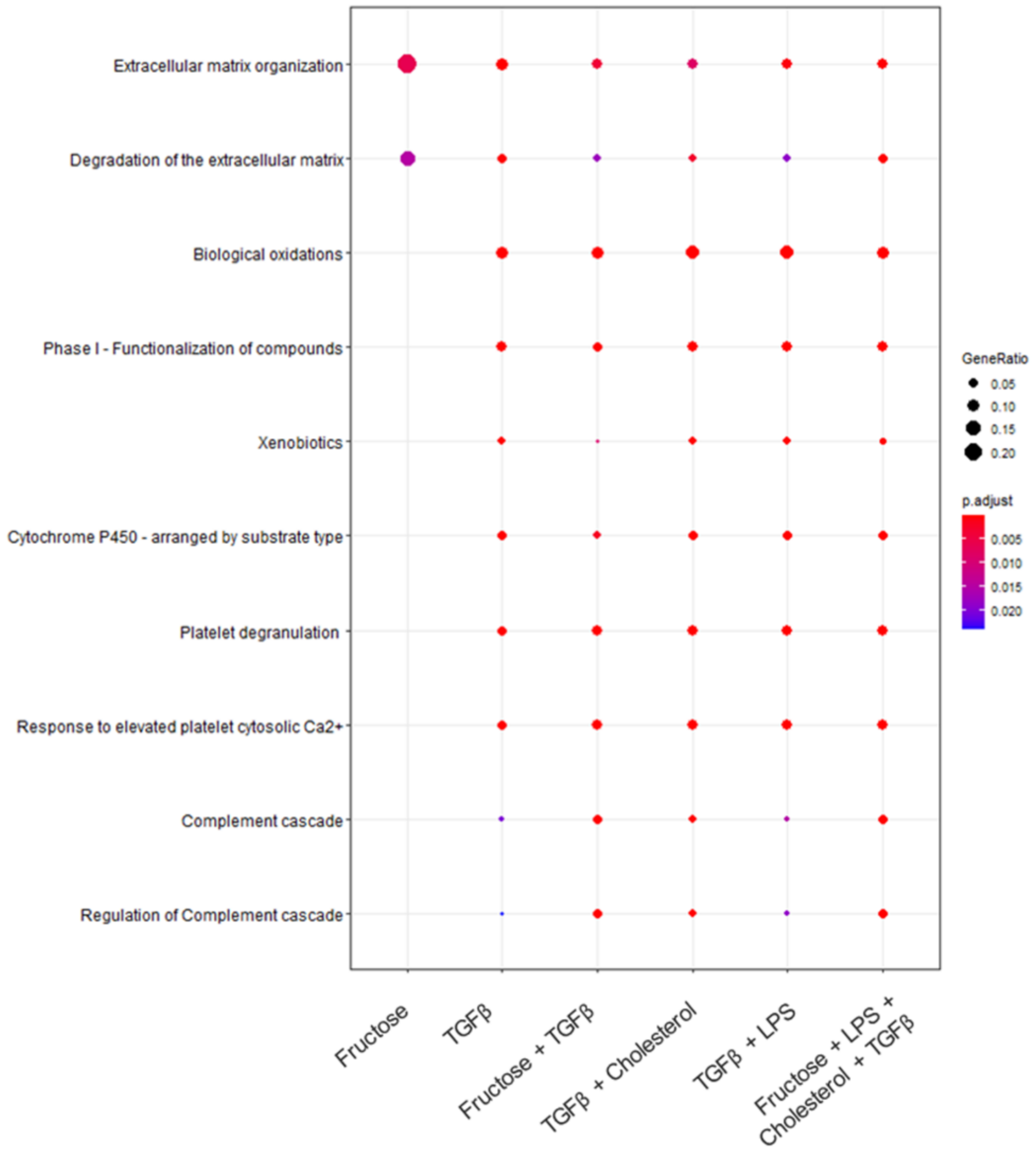

Supplemental Figure 8 – Transcriptional pathway analysis of DEGs in MPS NASH model with reduced NPC numbers following culture with varying disease associated cues.

PHH, KC and HSC co-cultures with reduced NPC numbers were cultured in the MPS platform in high fat media conditions for 14 days. Study was designed to compared effects of additional treatment with fructose, cholesterol, LPS and TGFβ (and combinations thereof) for effects on the transcriptional profile of the liver MPS NASH model. Total RNA was extracted from all samples after culture and compared by RNA-seq. Differentially expressed genes from varying conditions were compared to identify enriched pathways and biological processes, using PANTHER database and mapped using CompareCluster, with size of cluster representing number of genes involved and colour identifies confidence interval. Conditions that did not have any clustering of DEGs are not shown (Cholesterol, LPS, LPS + Fructose, LPS + Cholesterol, Fructose + Cholesterol). Transcriptomic data was generated from a minimum of three independent replicate samples per condition.

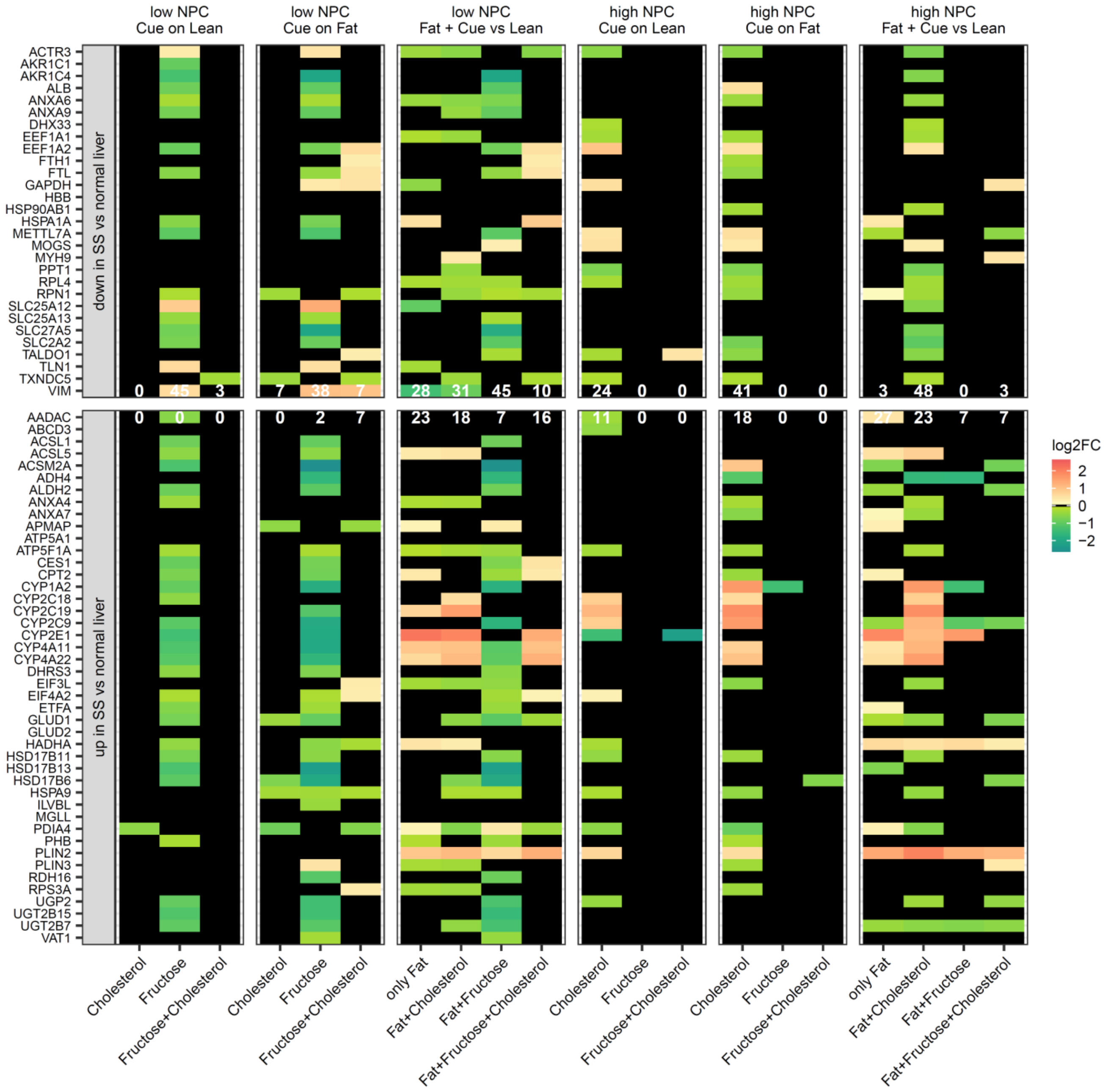

Supplemental Figure 9 – Assessment of lipid-droplet associated genes in MPS NASH model following culture with varying disease associated cues.

List of lipid-droplet associated genes that are differentially expressed in simple steatosis (SS) vs control human liver was obtained from literature[43]. The markers were used to compare gene expression between conditions with varying cues. PHH, KC and HSC co-cultures were cultured in the MPS platform under a variety of conditions for 14 days. Study was designed to test number of NPCs, presence of fat, fructose and cholesterol for effects on the transcriptional profile of the liver MPS NASH model. Total RNA was extracted from all samples after culture and compared by RNA-seq. The plot shows log fold change in the treated condition with the cues vs the corresponding control without cues. The white numbers indicate percentage of markers whose directionality of expression change matches expression changes observed in patients. Transcriptomic data was generated from a minimum of three independent replicate samples per condition.

Kostrzewski et al. 2021
COMMSBIO-21-0724B

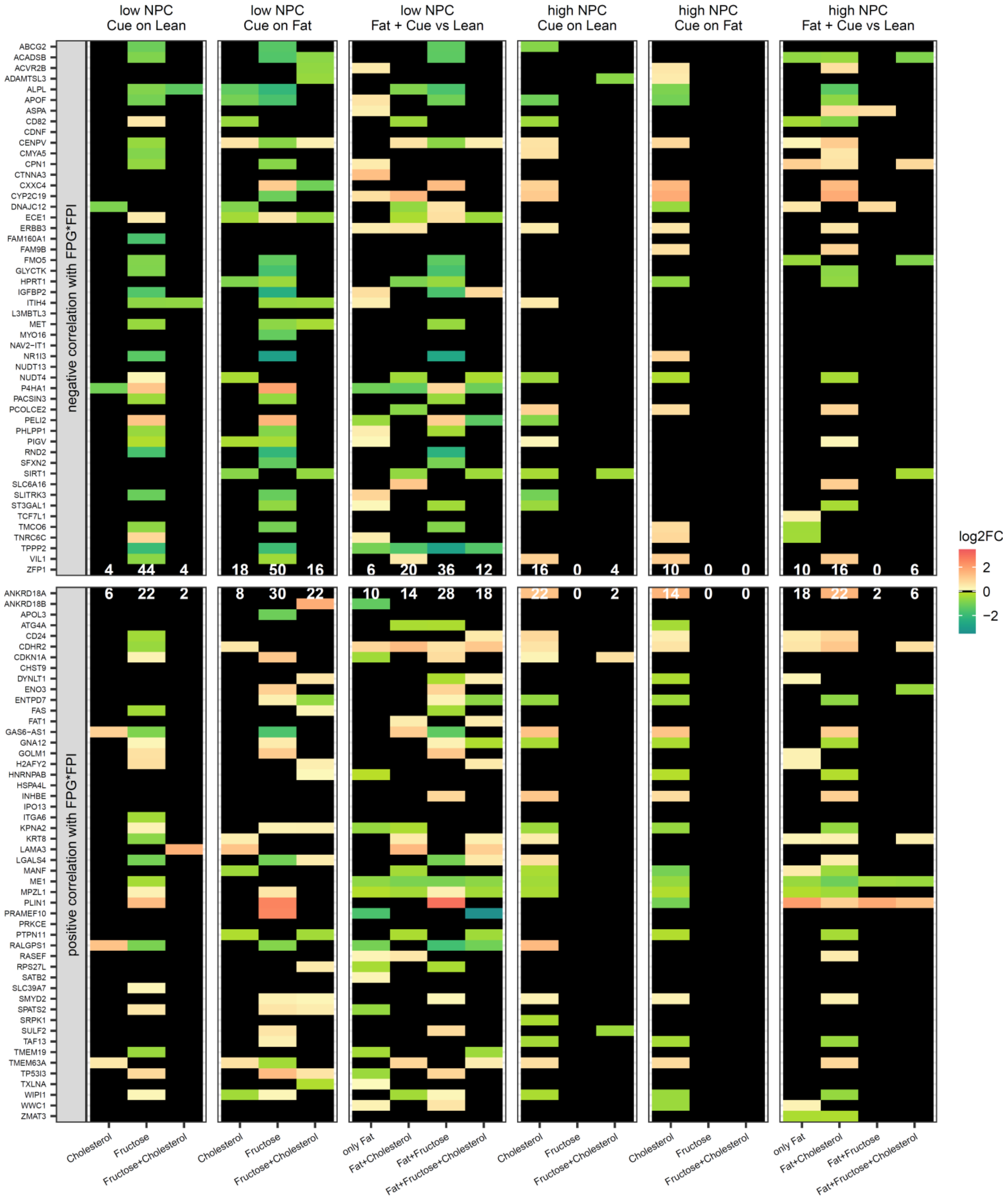

Supplemental Figure 10 – Assessment of insulin resistance markers in MPS NASH model following culture with varying disease associated cues.

Markers of hepatic insulin resistance were identified as top genes correlated to fasting plasma glucose x fasting blood insulin (FPG x FPI) in morbidly obese patients without statin treatment[44]. The markers were used to compare gene expression between conditions with varying cues. PHH, KC and HSC co-cultures were cultured in the MPS platform under a variety of conditions for 14 days. Study was designed to test number of NPCs, presence of fat, fructose and cholesterol for effects on the transcriptional profile of the liver MPS NASH model. Total RNA was extracted from all samples after culture and compared by RNA-seq. The plot shows log fold change in the treated condition with the cues vs the corresponding control without cues. The white numbers indicate percentage of markers whose directionality of expression change matches expression changes observed in patients. Transcriptomic data was generated from a minimum of three independent replicate samples per condition.

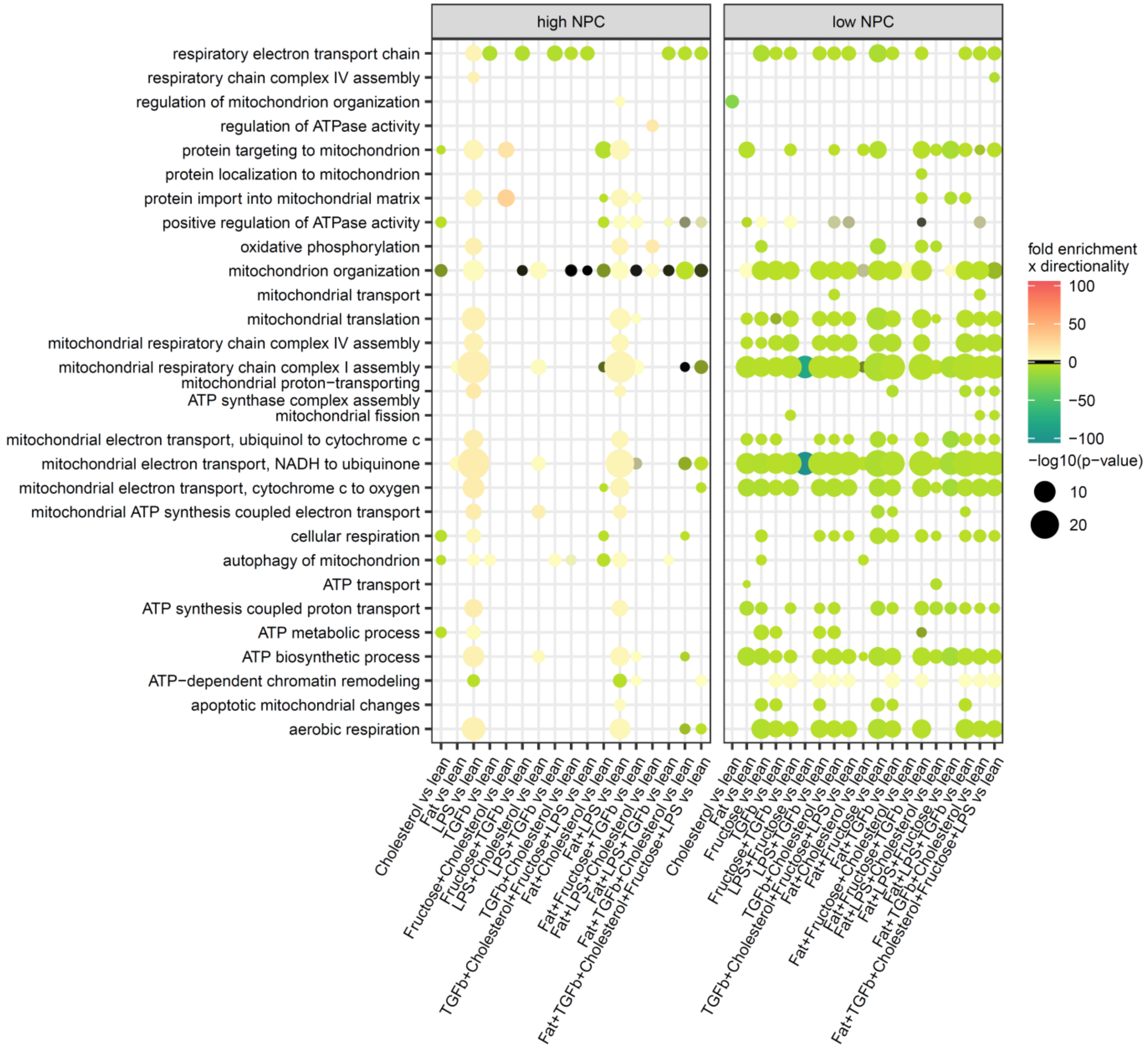

Supplemental Figure 11 – Assessment of GO biological process terms associated with mitochondrial function in MPS NASH model following culture with varying disease associated cues.

PHH, KC and HSC co-cultures were cultured in the MPS platform under a variety of conditions for 14 days. Study was designed to test number of NPCs, presence of cues and combination of cues for effects on the transcriptional profile of the liver MPS NASH model. Total RNA was extracted from all samples after culture and compared by RNA-seq. The plot shows GO biological processes associated with mitochondrial function for each cue or combination of cues compared to corresponding control without cues. Only GO terms reaching FDR < 0.05 in the respective contrasts are plotted. Size of dots represents number of transcripts associated with process and colour changes show direction of change. Transcriptomic data was generated from a minimum of three independent replicate samples per condition.

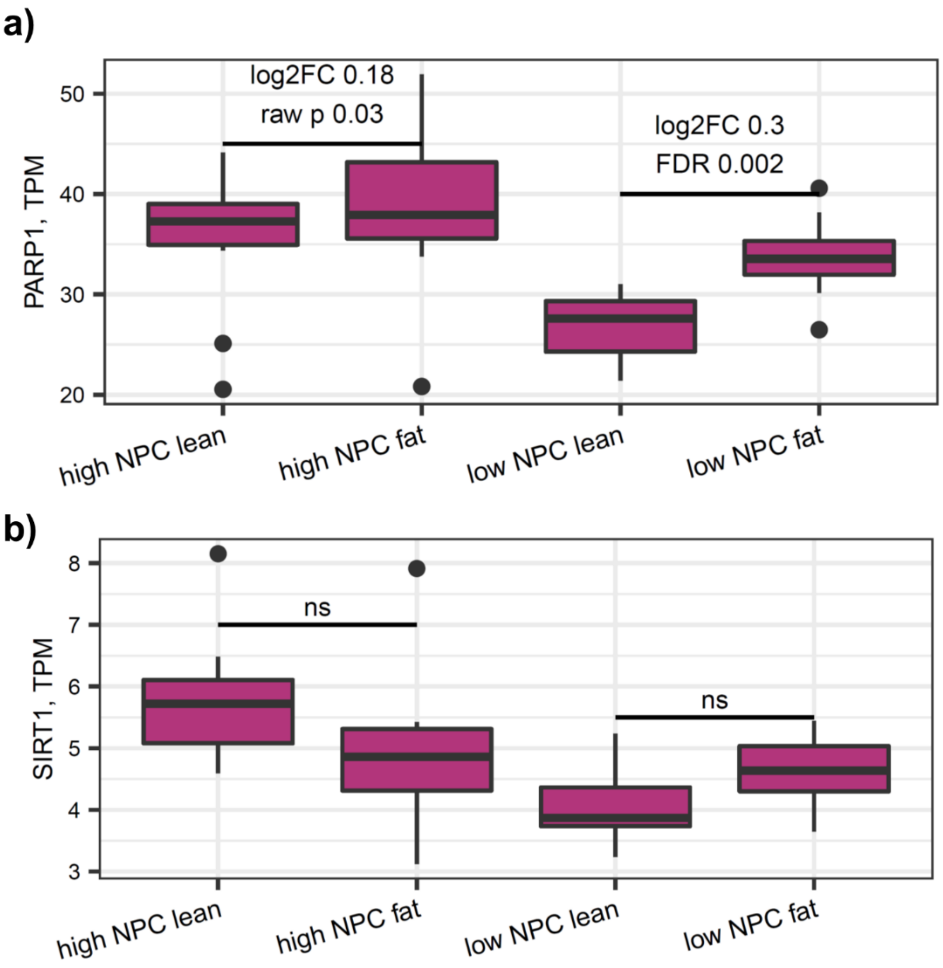

Supplemental Figure 12 – Assessment of SIRT-1 and PARP1 expression in MPS NASH model.

PHH, KC and HSC co-cultures were cultured in the MPS platform under a variety of conditions for 14 days. Total RNA was extracted from all samples after culture and compared by RNA-seq. A) PARP1 expression and B) SIRT1 expression in control samples from MPS. Transcriptomic data was generated from a minimum of nine independent replicate samples per condition. Plots show mean ± SD.

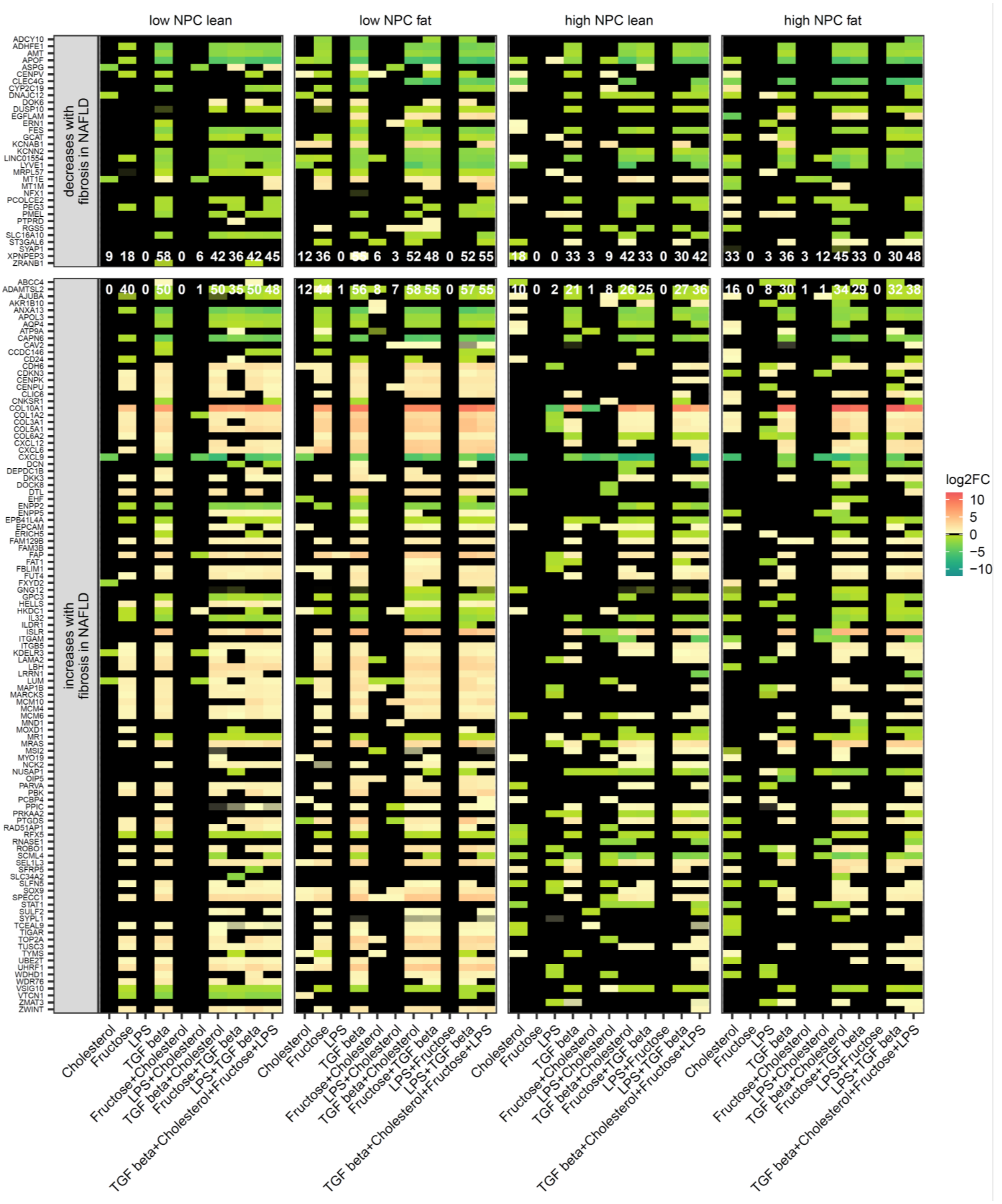
low NPC lean
low NPC fat
high NPC lean
high NPC fat
decreases with fibrosis in NAFLD
increases with fibrosis in NAFLD
log2FC
10
5
0
−5
−10
Cholesterol
Fructose
LPS
TGF beta
Fructose+Cholesterol
LPS+Cholesterol
TGF beta+Cholesterol
Fructose+TGF beta
LPS+Fructose
LPS+TGF beta
TGF beta+Cholesterol+Fructose+LPS

Supplemental Figure 13 – Assessment of fibrosis progression markers in MPS NASH model following culture with varying disease associated cues.

Subset of markers associated with fibrosis stage progression was extracted from meta-analysis[47]. The markers were used to compare gene expression between conditions with varying cues. PHH, KC and HSC co-cultures were cultured in the MPS platform under a variety of conditions for 14 days. Study was designed to test number of NPCs, presence of cues and combination of cues for effects on the transcriptional profile of the liver MPS NASH model. Total RNA was extracted from all samples after culture and compared by RNA-seq. The plot shows log fold change in the treated condition with the cues vs the corresponding control without cues. The white numbers indicate percentage of markers whose directionality of expression change matches expression changes observed in patients. Transcriptomic data was generated from a minimum of three independent replicate samples per condition.

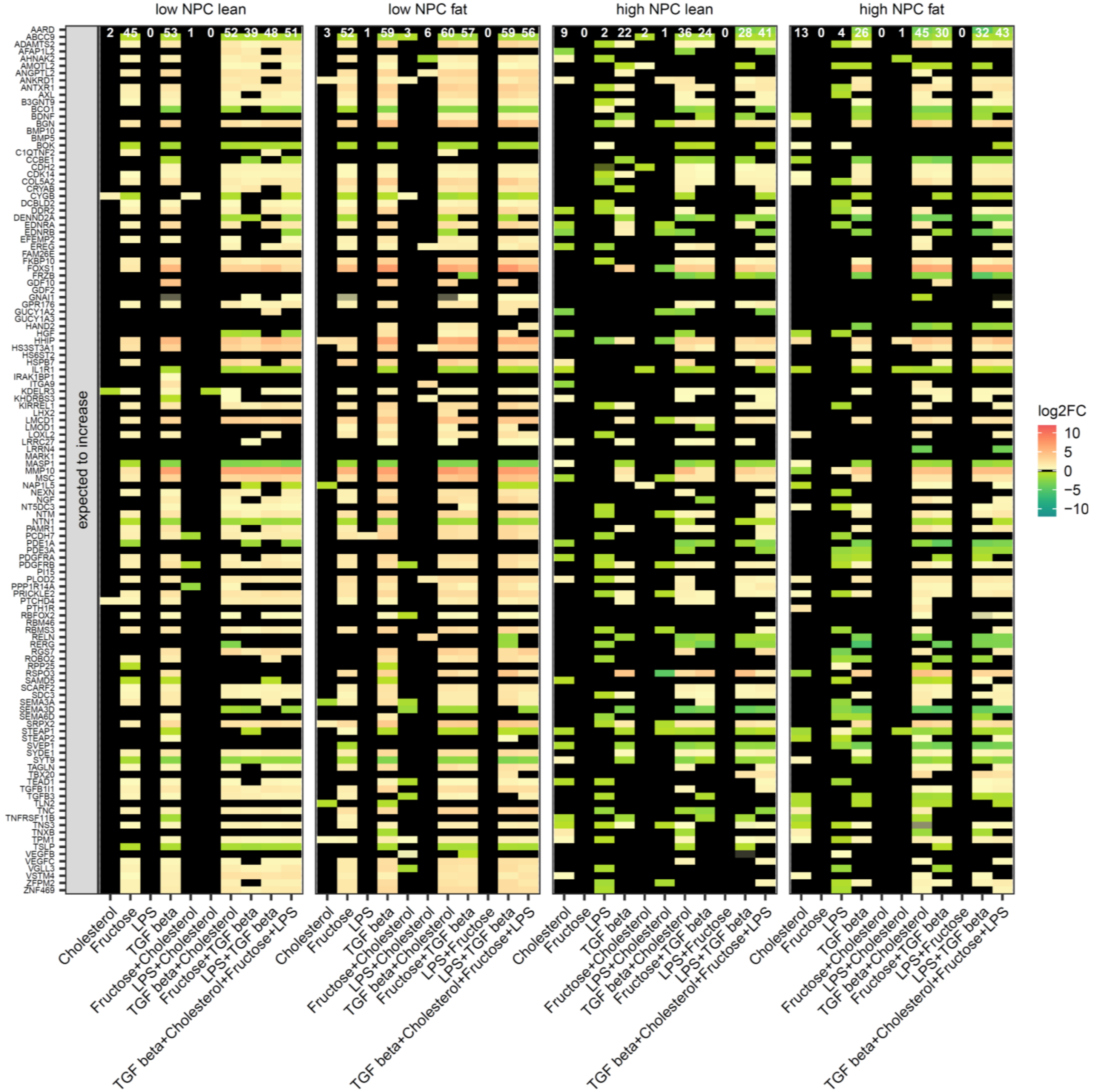

Supplemental Figure 14 – Stellate cell signature in MPS NASH model following culture with varying disease associated cues.

Stellate cell signature associated with NAFLD fibrosis were extracted from literature[48]. The markers were used to compare gene expression between conditions with varying cues. PHH, KC and HSC co-cultures were cultured in the MPS platform under a variety of conditions for 14 days. Study was designed to test number of NPCs, presence of cues and combination of cues for effects on the transcriptional profile of the liver MPS NASH model. Total RNA was extracted from all samples after culture and compared by RNA-seq. The plot shows log fold change in the treated condition with the cues vs the corresponding control without cues. The white numbers indicate percentage of markers whose directionality of expression change matches expression changes observed in patients. Transcriptomic data was generated from a minimum of three independent replicate samples per condition.

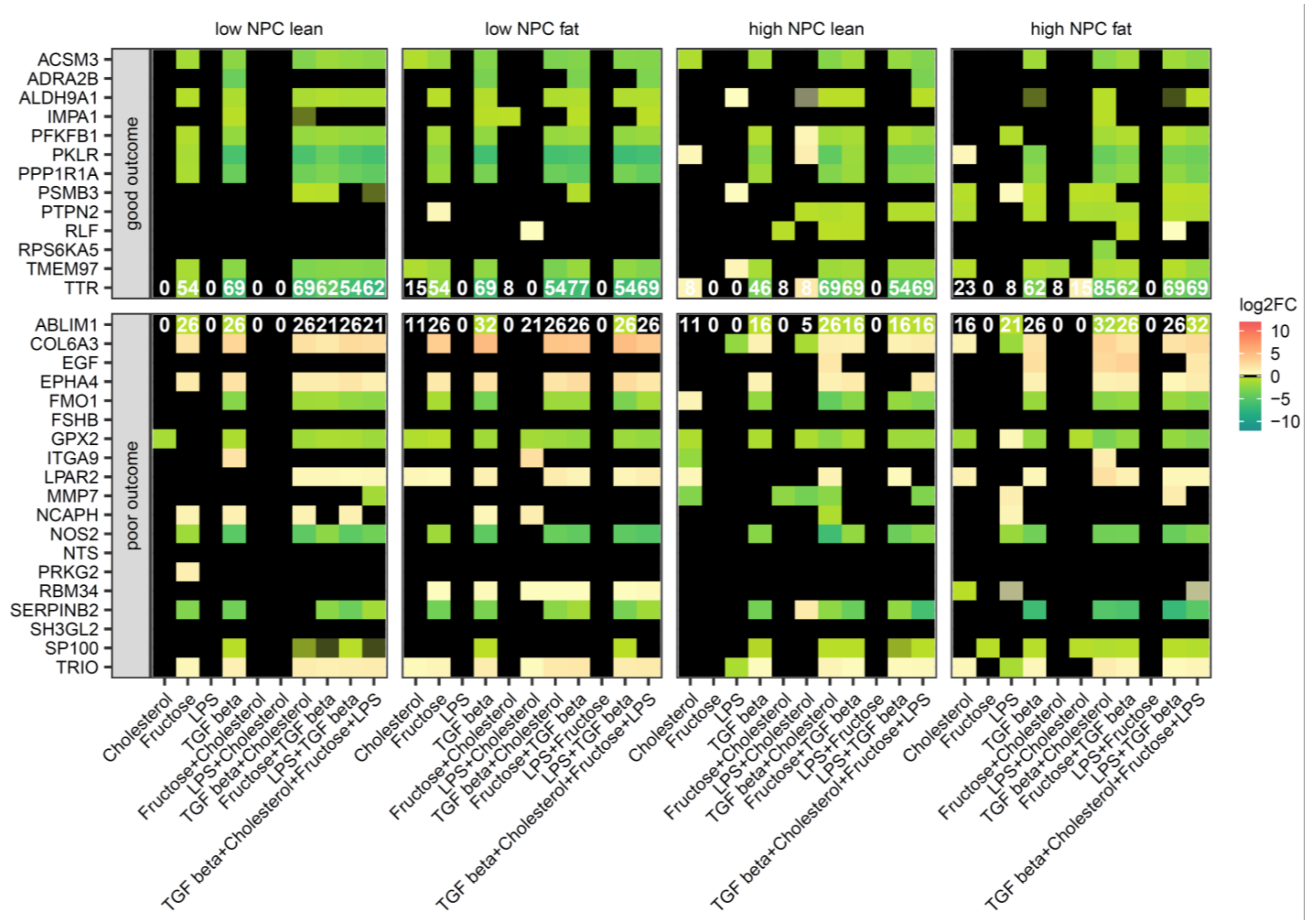

Supplemental Figure 15 – Clinical outcome markers in MPS NASH model following culture with varying disease associated cues.

32-gene signature associated with adverse clinical outcomes after bariatric surgery in NASH and HCC risk[49,50]. The markers were used to compare gene expression between conditions with varying cues. PHH, KC and HSC co-cultures were cultured in the MPS platform under a variety of conditions for 14 days. Study was designed to test number of NPCs, presence of cues and combination of cues for effects on the transcriptional profile of the liver MPS NASH model. Total RNA was extracted from all samples after culture and compared by RNA-seq. The plot shows log fold change in the treated condition with the cues vs the corresponding control without cues. The white numbers indicate percentage of markers whose directionality of expression change matches expression changes observed in patients. Transcriptomic data was generated from a minimum of three independent replicate samples per condition.

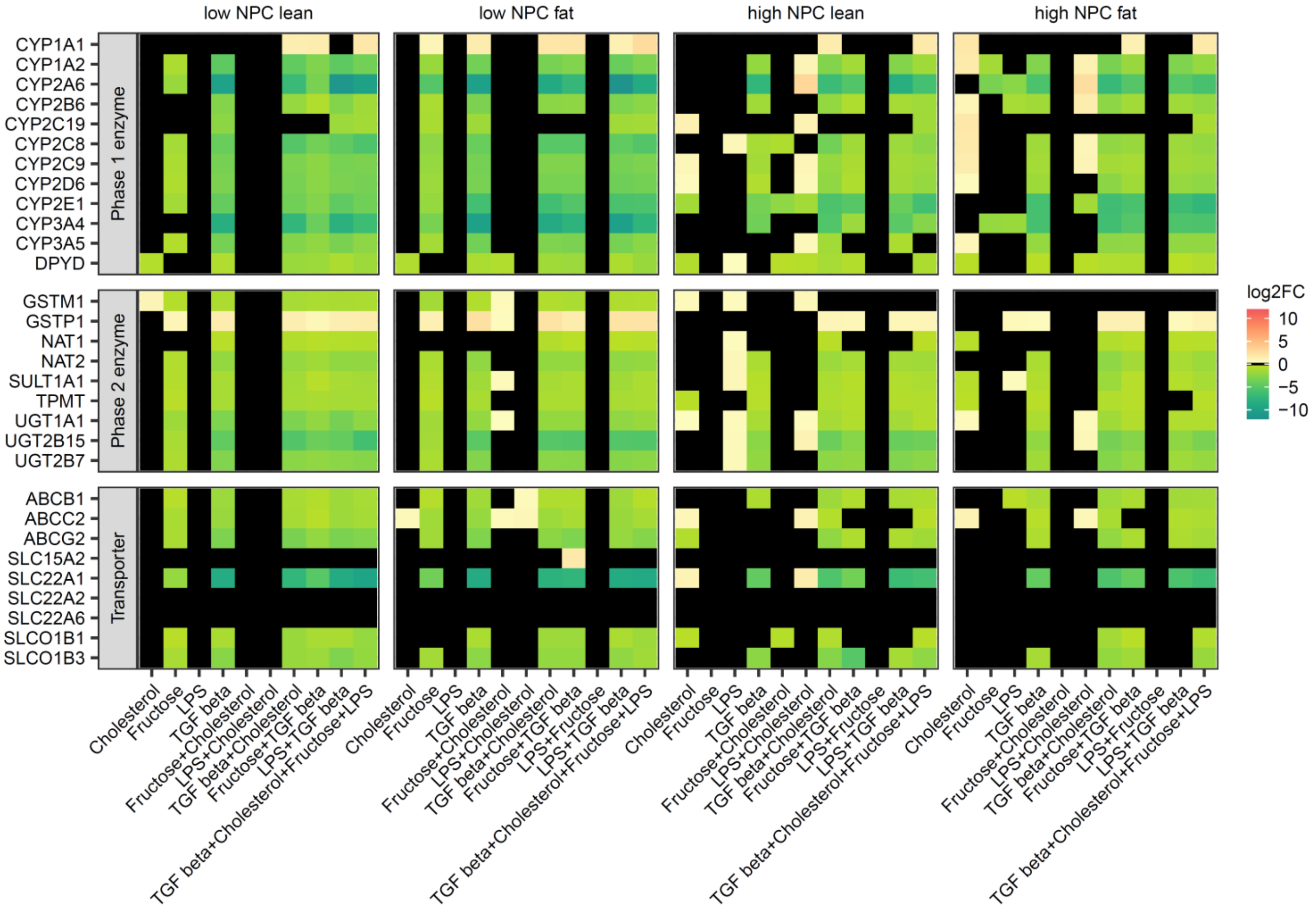

Supplemental Figure 16 – ADME genes in MPS NASH model following culture with varying disease associated cues.

Expression of core ADME genes were extracted from literature[51]. The markers were used to compare gene expression between conditions with varying cues. PHH, KC and HSC co-cultures were cultured in the MPS platform under a variety of conditions for 14 days. Study was designed to test number of NPCs, presence of cues and combination of cues for effects on the transcriptional profile of the liver MPS NASH model. Total RNA was extracted from all samples after culture and compared by RNA-seq. The plot shows log fold change in the treated condition with the cues vs the corresponding control without cues. The white numbers indicate percentage of markers whose directionality of expression change matches expression changes observed in patients. Transcriptomic data was generated from a minimum of three independent replicate samples per condition.

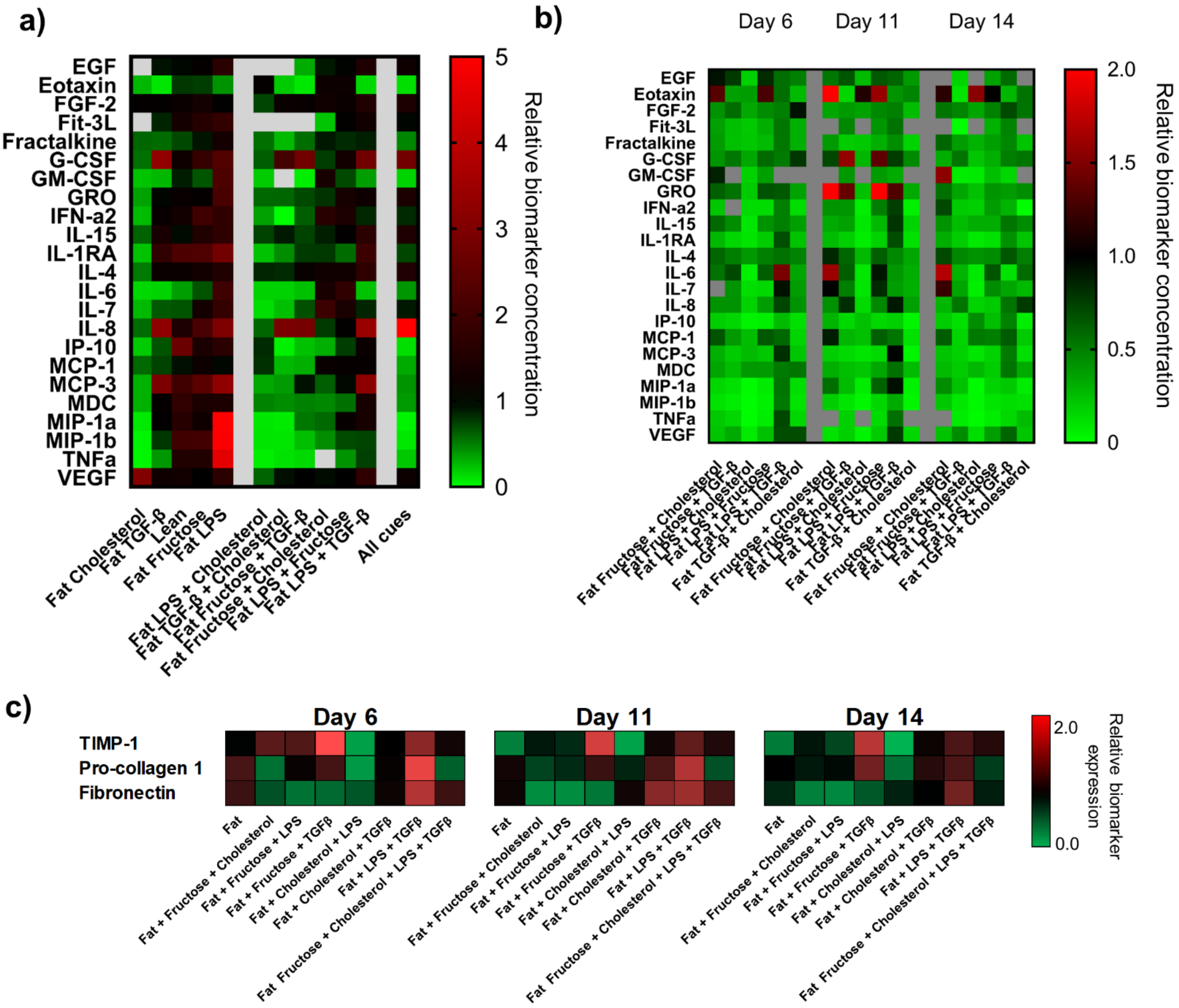

Supplemental Figure 17 – Synergistic or additive effects of biological cues on soluble biomarkers and cytokines in MPS NASH model.

PHH, KC and HSC co-cultures were cultured in the MPS platform for 14 days to test effect of fat, fructose, cholesterol, LPS and TGFβ (and combinations thereof) on the soluble biomarker profile of the liver MPS NASH model. A) Relative expression of inflammatory cytokines were compared across conditions, all cytokine concentrations were determined by Luminex profiling and normalised using Z-transformation for comparison (white = not detected). B) All multi-cue conditions were compared across three timepoints – day 6, 11, 14 for relative expression of each cytokine. Concentrations of biomarkers are normalised using Z-transformation. C) Pro-fibrotic biomarkers were compared using the same approach, these were measured by ELISA at three timepoints and normalised using Z-transformation. All data was generated from a minimum of three independent replicate samples per condition.

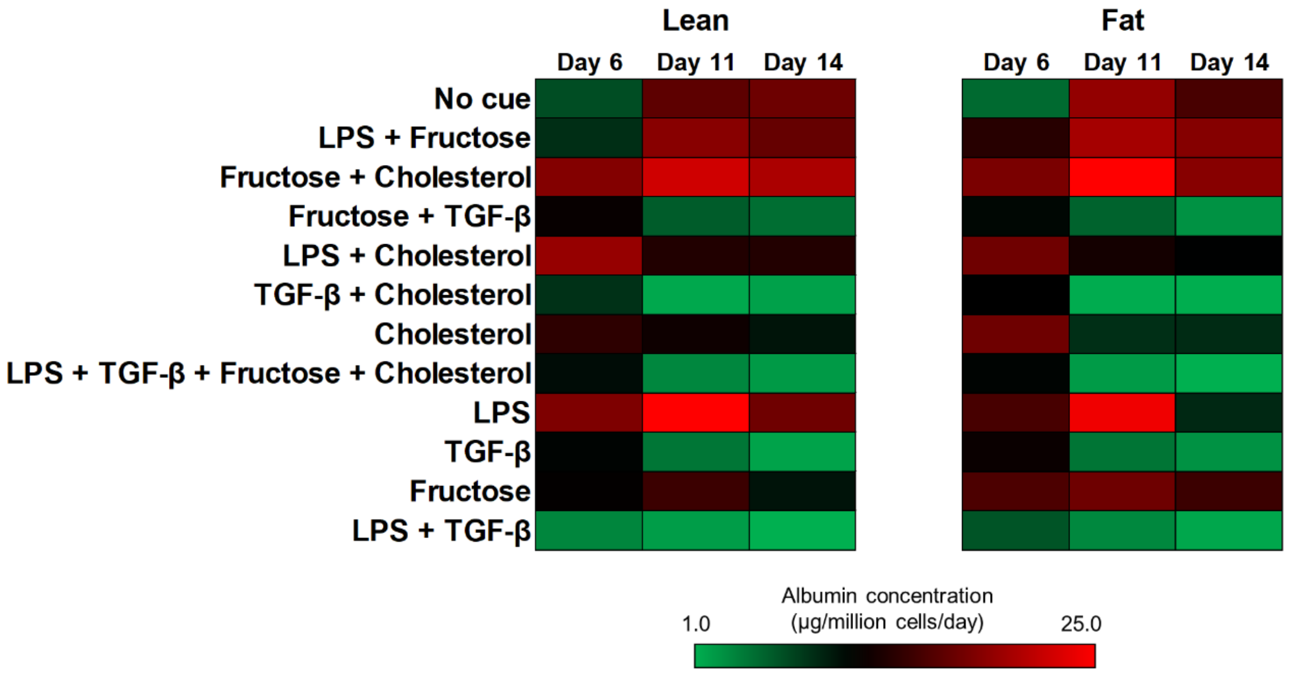

Supplemental Figure 18 – Effects of biological cues on albumin production in MPS NASH model cultured in HEP-Lean or HEP-Fat media.

PHH, KC and HSC co-cultures were cultured in the MPS platform for 14 days to test effect of fat, fructose, cholesterol, LPS and TGFβ on the albumin production of the liver MPS NASH model. Relative expression of human albumin was compared across conditions, all concentrations were determined by ELISA profiling. All data was generated from a minimum of three independent replicate samples per condition.

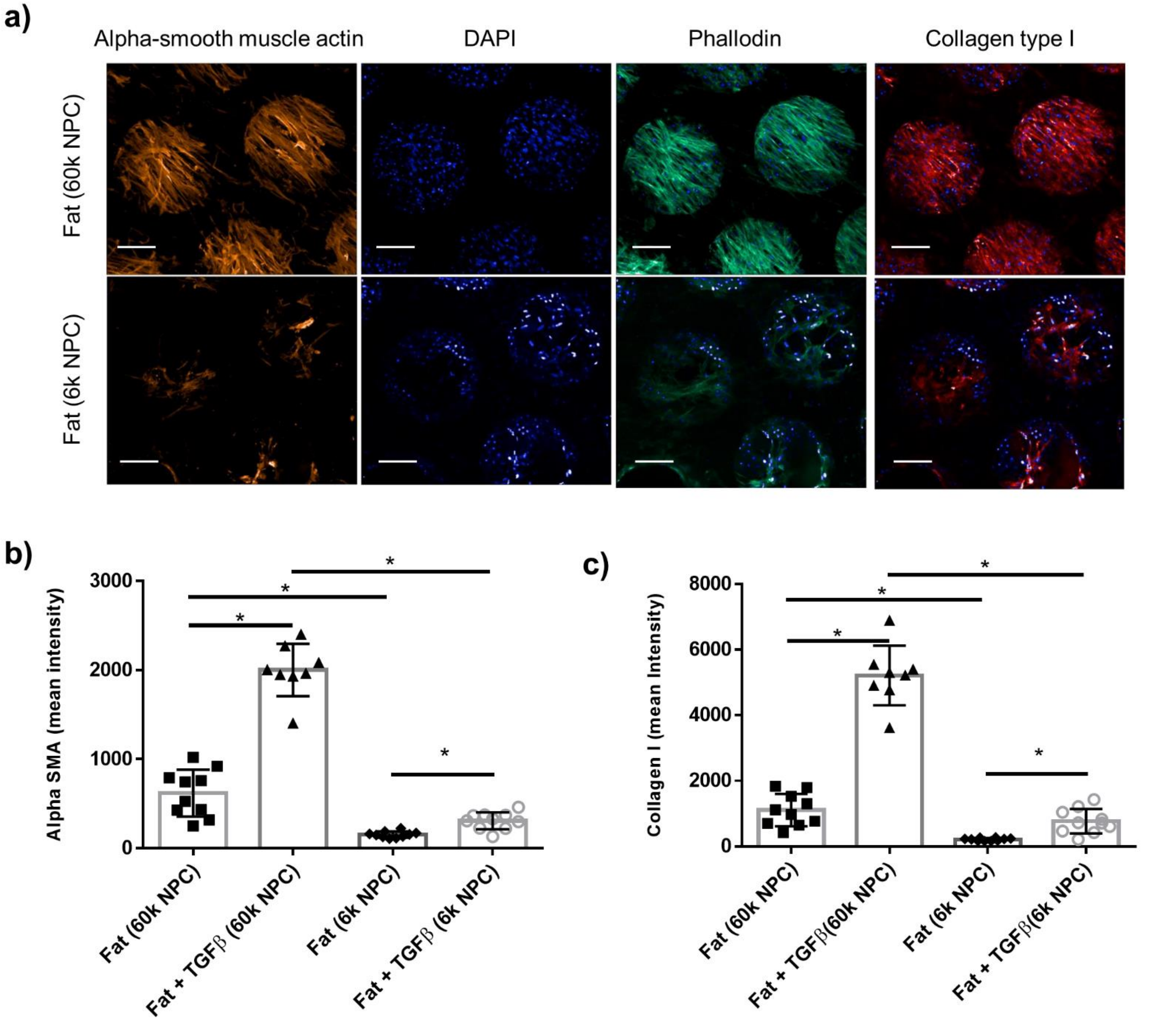

Supplemental Figure 19 – Reduced NPC levels significantly reduce fibrosis endpoints in MPS NASH model cultured in HEP-Fat media.

PHH, KC and HSC co-cultures with 60k or 6k NPCs were cultured in the MPS platform for 15 days in HEP-fat media or HEP-fat media + TGFβ. Liver microtissues were stained for cytoskeleton (phalloidin), collagen-type I and α-SMA and imaged by confocal microscopy. A) Representative images shown microtissues from HEP-fat media condition and scale bars 200 µm. B) Staining of microtissue was quantified by measuring total fluorescence intensity throughout individual microtissues, each data point represents an average of all microtissues within an MPS culture (min 8, max 20). All datapoints shown and error bars highlight mean ± SD. Data from a minimum of six biological replicates. P * < 0.05 determined by student T-test.
