# Supplementary material for: Modelling human liver fibrosis in the context of non-alcoholic steatohepatitis using a microphysiological system
Source: Commun Biol. 2021 Sep 15;4:1080. doi: 10.1038/s42003-021-02616-x (PMC8443589; doi:10.1038/s42003-021-02616-x)
Supplement: Supplementary file 3 — Description of Supplementary Files [file 42003_2021_2616_MOESM3_ESM.pdf]

Description of Additional Supplementary Files

File name: Supplementary Data 1

Description: Source data for main figures.
