# Supplementary material for: Modelling human liver fibrosis in the context of non-alcoholic steatohepatitis using a microphysiological system
Source: Commun Biol. 2021 Sep 15;4:1080. doi: 10.1038/s42003-021-02616-x (PMC8443589; doi:10.1038/s42003-021-02616-x)
Supplement: Supplementary file 5 — Reporting Summary [file 42003_2021_2616_MOESM5_ESM.pdf]

nature research

Corresponding author(s): Tomasz Kostrzewski

Last updated by author(s): Jul 30, 2021



# Reporting Summary

Nature Research wishes to improve the reproducibility of the work that we publish. This form provides structure for consistency and transparency in reporting. For further information on Nature Research policies, see our Editorial Policies and the Editorial Policy Checklist.

## Statistics

For all statistical analyses, confirm that the following items are present in the figure legend, table legend, main text, or Methods section.

| n/a | Confirmed | |
|---|---|---|
| ☐ | ☒ | The exact sample size ($n$) for each experimental group/condition, given as a discrete number and unit of measurement |
| ☐ | ☒ | A statement on whether measurements were taken from distinct samples or whether the same sample was measured repeatedly |
| ☐ | ☒ | The statistical test(s) used AND whether they are one- or two-sided<br>*Only common tests should be described solely by name; describe more complex techniques in the Methods section.* |
| ☐ | ☒ | A description of all covariates tested |
| ☐ | ☒ | A description of any assumptions or corrections, such as tests of normality and adjustment for multiple comparisons |
| ☐ | ☒ | A full description of the statistical parameters including central tendency (e.g. means) or other basic estimates (e.g. regression coefficient) AND variation (e.g. standard deviation) or associated estimates of uncertainty (e.g. confidence intervals) |
| ☐ | ☒ | For null hypothesis testing, the test statistic (e.g. $F$, $t$, $r$) with confidence intervals, effect sizes, degrees of freedom and $P$ value noted<br>*Give P values as exact values whenever suitable.* |
| ☒ | ☐ | For Bayesian analysis, information on the choice of priors and Markov chain Monte Carlo settings |
| ☒ | ☐ | For hierarchical and complex designs, identification of the appropriate level for tests and full reporting of outcomes |
| ☒ | ☐ | Estimates of effect sizes (e.g. Cohen's $d$, Pearson's $r$), indicating how they were calculated |

*Our web collection on statistics for biologists contains articles on many of the points above.*

## Software and code

Policy information about availability of computer code

| | |
|---|---|
| Data collection | No software code used |
| Data analysis | No software code used |

For manuscripts utilizing custom algorithms or software that are central to the research but not yet described in published literature, software must be made available to editors and reviewers. We strongly encourage code deposition in a community repository (e.g. GitHub). See the Nature Research guidelines for submitting code & software for further information.

## Data

Policy information about availability of data

All manuscripts must include a data availability statement. This statement should provide the following information, where applicable:

- Accession codes, unique identifiers, or web links for publicly available datasets
- A list of figures that have associated raw data
- A description of any restrictions on data availability

Transcriptomic data is deposited on GEO with accession number: GSE168285. Raw data is available for Figures 1-6 and Supplemental Figures 2-19 on request.

# Field-specific reporting

# Life sciences study design

All studies must disclose on these points even when the disclosure is negative.

| | |
|---|---|
| Sample size | All experiments were performed with a minimum of three independent liver MPS cultures, for the systems biology experiments control samples were performed with twelve indepedent liver MPS cultures to increase sample size |
| Data exclusions | No data was excluded from analysis. |
| Replication | Variation in all data sets collected is shown in the presented data in the manuscript. In the early part of the manuscript studies were performed with greater sample size and using multiple donor sources to show reprodubility across experiments for all important endpoints. For the main systems biology experiment it was not possible to repeat multiple times due to the size and complexity, but additional control samples were used to ensure reproducbility. The control samples were compared across multple metrics to ensure acceptable levels of variabilty before analysis of other conditions. |
| Randomization | When liver MPS cultures were generated with different culture conditions these we randomly distributed across MPS plates to reduce any plate specific effects. |
| Blinding | No blinding was relevant to this study. |

# Reporting for specific materials, systems and methods

We require information from authors about some types of materials, experimental systems and methods used in many studies. Here, indicate whether each material, system or method listed is relevant to your study. If you are not sure if a list item applies to your research, read the appropriate section before selecting a response.

## Materials & experimental systems

| n/a | Involved in the study |
|---|---|
| ☐ | ☒ Antibodies |
| ☒ | ☐ Eukaryotic cell lines |
| ☒ | ☐ Palaeontology and archaeology |
| ☒ | ☐ Animals and other organisms |
| ☒ | ☐ Human research participants |
| ☒ | ☐ Clinical data |
| ☒ | ☐ Dual use research of concern |

## Methods

| n/a | Involved in the study |
|---|---|
| ☒ | ☐ ChIP-seq |
| ☒ | ☐ Flow cytometry |
| ☒ | ☐ MRI-based neuroimaging |

## Antibodies

Antibodies used

- anti- collagen type 1 (Rabbit polyclonal). (Rockland, USA) #600 401 103 0.5
- anti-alpha smooth muscle Actin antibody [1A4]. (Abcam, UK). #ab7817
- Phalloidin 488 (Thermofisher, UK) #A12379
- Alexa-Fluor 555 goat anti-rabbit. (Thermofisher, UK). #A21429
- Alexa-Fluor 647 donkey anti-rabbit. (Thermofisher, UK). #A31573
- Alexa-Fluor 555 goat anti-mouse. (Thermofisher, UK). #A21424
- Alexa-Fluor 647 goat anti-mouse (Thermofisher, UK). #A21235

Validation

Validation of alpha smooth muscel antibody can be found on manufactures website: https://www.abcam.com/alpha-smooth-muscle-actin-antibody-1a4-ab7817.html
Validation for collagen type 1 antibody available on manufactures website: https://rockland-inc.com/store/Extracellular-Matrix-Antibodies-600-401-103-0.5-O4L_24102.aspx
